# Introductions and early spread of SARS-CoV-2 in the New York City area

**DOI:** 10.1126/science.abc1917

**Published:** 2020-05-29

**Authors:** Ana S. Gonzalez-Reiche, Matthew M. Hernandez, Mitchell J. Sullivan, Brianne Ciferri, Hala Alshammary, Ajay Obla, Shelcie Fabre, Giulio Kleiner, Jose Polanco, Zenab Khan, Bremy Alburquerque, Adriana van de Guchte, Jayeeta Dutta, Nancy Francoeur, Betsaida Salom Melo, Irina Oussenko, Gintaras Deikus, Juan Soto, Shwetha Hara Sridhar, Ying-Chih Wang, Kathryn Twyman, Andrew Kasarskis, Deena R. Altman, Melissa Smith, Robert Sebra, Judith Aberg, Florian Krammer, Adolfo García-Sastre, Marta Luksza, Gopi Patel, Alberto Paniz-Mondolfi, Melissa Gitman, Emilia Mia Sordillo, Viviana Simon, Harm van Bakel

**Affiliations:** 1 Department of Genetics and Genomic Sciences, Icahn School of Medicine at Mount Sinai, New York, NY 10029, USA.; 2 Department of Microbiology, Icahn School of Medicine at Mount Sinai, New York, NY 10029, USA.; 3 The Graduate School of Biomedical Sciences, Icahn School of Medicine at Mount Sinai, New York, NY 10029, USA.; 4 Clinical Microbiology Laboratory, Department of Pathology, Molecular, and Cell-Based Medicine, Icahn School of Medicine at Mount Sinai, New York, NY 10029, USA.; 5 Icahn Institute for Data Science and Genomic Technology, Icahn School of Medicine at Mount Sinai, New York, NY 10029, USA.; 6 The Mount Sinai Data Office, Mount Sinai Health System, New York, NY 10029, USA.; 7 Division of Infectious Diseases, Department of Medicine, Icahn School of Medicine at Mount Sinai, New York, NY 10029, USA.; 8 Black Family Stem Cell Institute, Icahn School of Medicine at Mount Sinai, New York, NY 10029, USA.; 9 Sema4, Stamford, CT 06902, USA.; 10 The Global Health and Emerging Pathogens Institute, Icahn School of Medicine at Mount Sinai, New York, NY 10029, USA.; 11 The Tisch Cancer Institute, Icahn School of Medicine at Mount Sinai, New York, NY 10029, USA.; 12 Department of Oncological Sciences, Icahn School of Medicine at Mount Sinai, New York, NY 10029, USA.

## Abstract

New York City (NYC) has emerged as one of the epicenters of the current severe acute respiratory syndrome coronavirus 2 (SARS-CoV-2) pandemic. To identify the early transmission events underlying the rapid spread of the virus in the NYC metropolitan area, we sequenced the virus that causes coronavirus disease 2019 (COVID-19) in patients seeking care at the Mount Sinai Health System. Phylogenetic analysis of 84 distinct SARS-CoV-2 genomes indicates multiple, independent, but isolated introductions mainly from Europe and other parts of the United States. Moreover, we found evidence for community transmission of SARS-CoV-2 as suggested by clusters of related viruses found in patients living in different neighborhoods of the city.

## Blighted Gotham

Deaths caused by severe acute respiratory syndrome coronavirus 2 (SARS-CoV-2) in New York City (NYC) during the spring of 2020 have vastly exceeded those reported in China and many other countries. What were the early events that led to such a severe outbreak? Gonzalez-Reiche *et al.* sampled some of the early patients seeking assistance in February and March of 2020 at the Mount Sinai Health System. Phylogenetic analysis of virus sequences in these people, who were drawn from across NYC, showed that the virus had been independently introduced many times from Europe and elsewhere in the United States. Subsequent clusters of community transmission occurred. The focus of infection in NYC is a marker of the role this city plays as a two-way hub for human movement.

*Science* this issue p. 297

## Teaser

New York City became an epicenter for COVID-19 cases that originated in Europe and elsewhere in the United States.

Severe acute respiratory syndrome coronavirus 2 (SARS-CoV-2; previously known as 2019-nCoV) is an emerging viral pathogen that was first reported to cause severe respiratory infections in Wuhan, China, in late December 2019. Over the past months, it rapidly spread across the globe, and the World Health Organization (WHO) declared a pandemic on 11 March 2020. Targeted screening of suspected coronavirus disease 2019 (COVID-19) cases, as well as a series of successive nationwide travel restrictions, were put in place to curtail SARS-CoV-2 introductions into the continental United States from outbreak hotspots in China (2 February 2020), Iran (2 March 2020), mainland European countries (13 March 2020), and the United Kingdom and Ireland (16 March 2020) (*1*–*4*). Despite these measures, the first COVID-19 case in New York State was identified in New York City (NYC) on 29 February 2020. During the first weeks of March, the number of detected cases rapidly increased because of expansion of screening capacity after implementation of automated platforms for detection of SARS-CoV-2 infections by local health system diagnostic laboratories and reference laboratories. As of 2 May 2020, there were 312,977 confirmed COVID-19 cases in New York state, including 172,354 (55%) in NYC [New York State Department of Health (NYS DOH); https://coronavirus.health.ny.gov]. With more than 13,300 fatalities in the metropolitan area, NYC has been one of the major epicenters of SARS-CoV-2 infections in the United States.

The Pathogen Surveillance Program (PSP) at the Icahn School of Medicine at Mount Sinai is a multidisciplinary, institutional infrastructure that seeks to generate high-resolution, near real-time genetic information on pathogens found to cause disease in the large and diverse patient population seeking care at the Mount Sinai Health System in NYC. After biospecimen coding, nucleic acid extraction, and polymerase chain reaction quantification, next-generation sequencing approaches based on Illumina and Pacific Biosciences technology provide information on the pathogen’s genome. The process has been optimized for quick turnaround, optimized data assembly, and integration with deidentified demographic information.

We took advantage of the existing PSP infrastructure to investigate the origins of SARS-CoV-2 strains circulating in NYC and to dissect the spread of the virus in this metropolitan area with a high-density population. Here, we present the genomic diversity of 90 SARS-CoV-2 isolates obtained from 84 patients seeking care at the Mount Sinai Health System between 29 February 2020 and 18 March 2020. These genomes provide clear evidence for multiple, independent SARS-CoV-2 introductions into NYC during the first weeks of March 2020. On the basis of genetic similarity and phylogenetic analysis of full-length viral genome sequences, most cases diagnosed during the 18 days after the first-reported COVID-19 case in New York state appear to be associated with untracked transmission and potential travel-related exposures. The majority of introductions appear to have been sourced from Europe and the United States. We also identified two clusters that total 21 closely related cases, suggesting community spread. These observations are also supported by the citywide distribution of these cases, which mapped to three of four represented NYC boroughs and five New York state counties. Our data point to the limited efficacy of travel restrictions into NYC for preventing spread in the metropolitan area once multiple introductions of SARS-CoV-2 and community-driven transmission had already occurred.

**Fig. 1:**
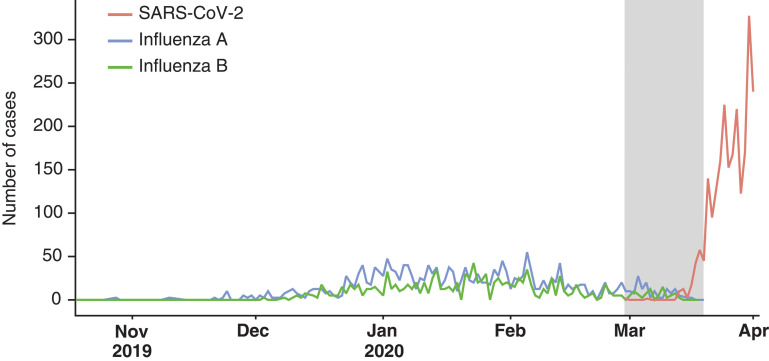
SARS-CoV-2 confirmed cases. Number of patients with positive molecular tests for SARS-CoV-2 up to 31 March 2020, compared with the daily number of patients with influenza A and B tests in the 2019–2020 season. The shaded area indicates the period in which the samples sequenced in this study were obtained (29 February to 18 March). The number of positive tests per virus was not normalized for the number of tested samples.

Initial diagnostic testing for SARS-CoV-2 in NYC was targeted and limited to individuals who fit a set of criteria outlined by the Centers for Disease Control and Prevention (CDC) and required preapproval by the local Department of Health. Between 9 March and 14 March 2020, screening capacity at the Mount Sinai Health System was greatly expanded, leading to a surge of newly diagnosed cases (Fig. 1). Within 1 week, the number of daily positive SARS-CoV-2 tests exceeded the normal volume of positive tests for influenza virus by a factor of 5. We sequenced 90 SARS-CoV-2 genomes from clinical isolates obtained from 84 of the more than 800 cases identified up to 18 March, yielding 72 complete and 18 partial (>95% coverage) genomes. These cases were drawn from 21 NYC neighborhoods across four boroughs (Manhattan, Bronx, Queens, and Brooklyn) as well as two towns in Westchester County. Sequenced isolates were obtained directly from nasopharyngeal swab samples collected from 44 females (52.4%) and 40 (47.7%) males ranging in age from 20 to 44 years (16%), 45 to 64 years (33%), 65 to 79 years (27.4%), and >80 years (16.7%). Of the 68 cases with available hospital visit information, 12 were discharged (17.6%), 53 were admitted (80%), and three were initially discharged and later admitted on a subsequent visit (2.4%).

**Fig. 2:**
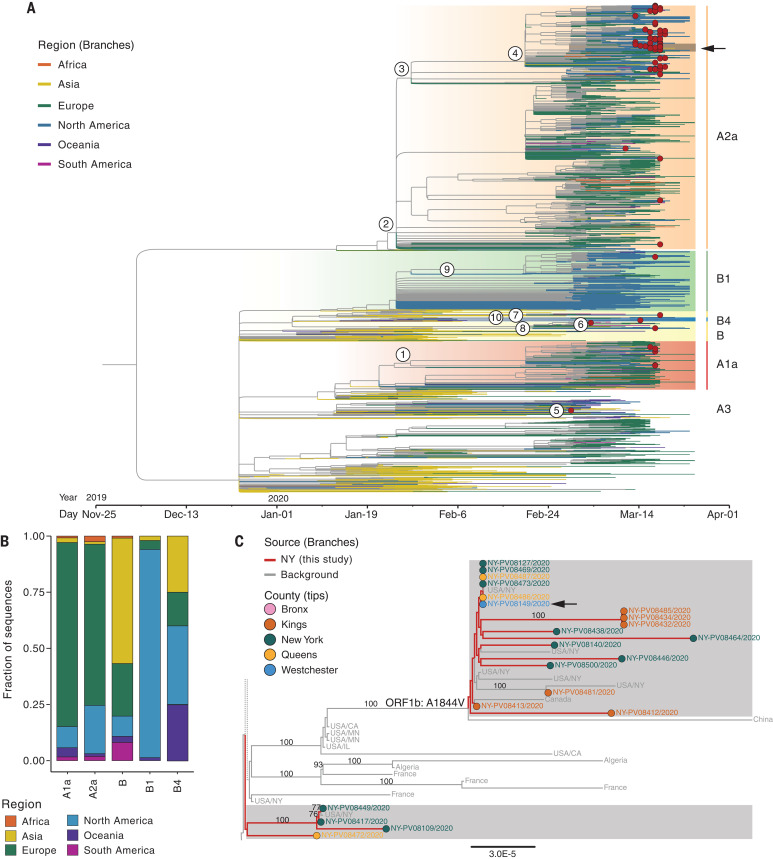
Phylogenetic relationships of SARS-CoV-2 from New York and other global strains. (**A**) ML phylodynamic inference of 84 SARS-CoV-2 sequences from New York from this study in a global background of 2363 sequences available in the GISAID EpiCoV database as of 1 April 2020. Branches are colored according to the region of origin. Tip circles (red) indicate the position of the 84 New York sequences. Clades that contain New York sequences are highlighted, with names shown on the right; the local transmission clusters are indicated by the arrow. The node positions for the transmission events listed in Table 1 are marked by the numbers in white circles. The displayed time tree was inferred under a strict clock model with a fixed substitution rate of 0.8 × 10^–3^. (**B**) Stacked barplot showing the fraction of sequences per region by clade. (**C**) Local transmission clusters on the ML tree showing the source of cases by county. Bootstrap support values ≥70% are shown; sibling clusters are collapsed for easier visualization. The mutation identified specific to the community transmission cluster is indicated. The scale bar at the bottom indicates the number of nucleotide substitutions per site.

We performed phylogenetic analysis of the 84 distinct patient isolates, together with 2363 sequences deposited in GISAID up to 1 April 2020 (Fig. 2A). NYC isolates were distributed throughout the phylogenetic tree, which is consistent with multiple independent introductions. We assigned each SARS-CoV-2 isolate derived from a patient seeking care at the Mount Sinai Health System to one of the main monophyletic clades on the basis of amino acid and nucleotide substitutions and statistical support from both maximum-likelihood (ML) and Bayesian methods. These mutations were used only for the purpose of classification because the functional impact of many of these substitutions remains unknown. As a reference, we adopted the clade nomenclature from the NextStrain tool (*5*, *6*). For each assigned clade, we identified different types of events according to the position of the NYC sequences and available epidemiological information (Table 1). The first isolate was obtained from a patient with documented exposure through travel to the Middle East (clade A3, node 5), and the second was obtained from a patient with documented travel to Europe (clade B, node 6). We therefore excluded these two cases from any inference made from the phylogenetic analyses. For the remaining isolates, the great majority (87%) clustered with clade A2a. This clade is largely composed of isolates obtained from patients with COVID-19 in Europe (72%) (Fig. 2B), suggesting that introductions from Europe account for the majority of cases found in NYC in the first weeks of March 2020.

**Table 1:** Inferred SARS-CoV-2 virus transmission events related to NYC.

**Clade**	**First NYC sequence**	**Mutations**	**Node**	**Cases**	**Event type**	**Inferred source**	tMRCA 95% HPD*
A1a	4 March	ORF3a:G251V, C14805T	1	5	Putative introduction	Europe	25 January to 6 February
A2a	11 March	S:D624G, ORF1b:P314L	2	4	Untracked transmission	N/A	5 January to 29 January
		S:D624G, ORF1b:P314L,OFR3a:Q57H	3	5	Putative introduction	Europe/North America	31 January to 11 February
		S:D624G, ORF1b:P314L,OFR3a:Q57H, ORF1a:T265I	4	64	Putative introduction	Europe/North America	15 February to 19 February
A3	29 February	ORF1a:V378I	5	1	Travel exposure	Middle East (epi link)	29 February (Test date)
B	4 March	ORF8:L84S	6	1	Travel exposure	Europe (epi link)	4 March (Test date)
			7	1	Putative introduction	Europe/Asia	2 February to 22 February
			8	1	Putative introduction	North America/Domestic	28 January to 24 February
B1	17 March	C18060T, ORFb:P1427L	9	1	Putative introduction	Domestic	26 January to 7 February
B4	14 March	N:S202N, ORF14:V94I	10	1	Putative introduction	Asia/Oceania	31 January to 25 February

*Inferred 95% High Posterior Density (HPD) interval for the mean time of tMRCA from the root of the node containing New York–origin strains with ≥70% bootstrap support value in the ML tree and ≥0.9 posterior probability in the maximum clade credibility (MCC) trees inferred for major clades. The 95% HPD intervals shown correspond to the MCC trees inferred by using a strict clock model. Travel exposures and putative introductions are shown for the cases described in this study.

Most of the NYC isolates within clade A2a were interspersed, and we did not observe grouping by country or geographical region (fig. S1). Despite their diverse origins, many sequences in this clade are highly similar or identical, which makes it impossible to resolve direct relationships or directionality between cases without additional epidemiological data. We next used ML and Bayesian phylodynamic analysis to infer the time of virus transmission events related to NYC (fig. S2A). For clade A2a, we estimated a period of untracked global transmission from late January to mid-February, which is consistent with epidemiological observations of the developing pandemic (Table 1, node 2, and fig. S2A). The earliest sequences at the base of clade A2a include isolates from Italy, Finland, Spain, France, the United Kingdom, and other European countries from late February, in addition to a few North American isolates (Canada and United States) from the first week of March 2020. Within this clade, we identified two mutations that distinguish clusters of sequences from NYC and elsewhere, suggesting at least two independent introductions that likely occurred in early to mid-February (Table 1, nodes 3 to 4, and fig. S2A). One of these two clusters contained 64 of the 72 NYC isolates positioned in clade A2a, suggesting local spread.

Similar to isolates in clade A2a, SARS-CoV-2 isolates in our study positioned in clade A1a (6%) were interspersed among isolates from multiple regions with unknown directionality (Fig. 2A, node 1). This clade is also largely composed of European-origin isolates (82%) (Fig. 2B and fig. S1).

For the rest of the clades (B, B1, and B4), we identified four putative SARS-CoV-2 virus introductions to NYC sometime between February and early March (Table 1, nodes 7 to 10, and fig. S2A). Two of these introductions were inferred to be of domestic origin on the basis of their close relationship with U.S. isolates, including those from the main community transmission in Washington state (clade B1, node 9) (*7*). The introduction of this clade to the East Coast was recently reported (*8*). Although more than half of the sequences in clade B were of Asian origin (Fig. 2B), the closest relatives to the New York isolates were of European and North American origin. The isolate that belongs to clade B4 is within a node with an inferred date of early March and a prior period of untracked transmission in unknown location(s) during February (Table 1, node 10, and fig. S2A). Before this period, the closest viral isolates basal to this cluster are from Australia and China (fig. S1).

**Fig. 3:**
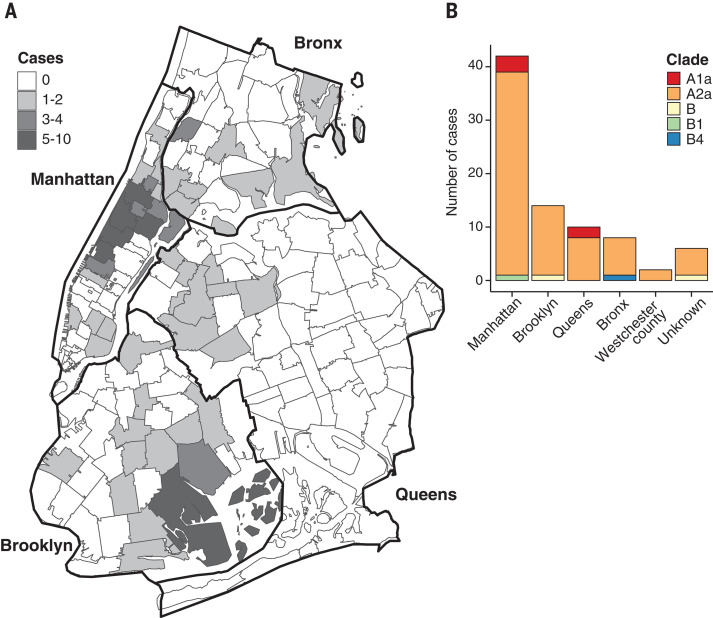
Distribution of the geographic location of the patients with COVID-19 from which viral isolates were sequenced. (**A**) Distribution of 74 sequenced cases with available ZIP code information across NYC boroughs and neighborhoods. Neighborhoods are shaded according to the number of cases that were sampled. (**B**) Breakdown of sequenced cases according to phylogenetic clades across NYC boroughs and Westchester County. Cases without available ZIP code information are indicated as “Unknown.”

The sequenced isolates and assigned clades were spatially distributed throughout all NYC boroughs and 21 neighborhoods (Fig. 3). Isolates sequenced at other NYC hospitals collected within a comparable time window had a similar clade and spatial distribution (*9*).

Taken together, our results show that the NYC SARS-CoV-2 epidemic has been mainly sourced from untracked transmission between the United States and Europe, with limited evidence of direct introductions from China, where the virus originated.

Despite the relatively small number of SARS-CoV-2 sequences available in early April 2020, we identified two monophyletic clusters positioned within clade A2a that almost exclusively contained isolates from New York (Fig. 2B). One cluster included 23% (17) of the isolates contained in clade A2a and 20% of the total isolates sequenced. According to ZIP code information, the cases from this cluster were distributed across five counties, including one sample from New Rochelle, Westchester County, which is part of the metropolitan area directly north of NYC and reported the first documented cluster of community-acquired infections in New York state on 3 March 2020. This cluster was characterized by the amino acid substitution A1844V in the ORF1b gene [in which alanine (A) was replaced at position 1844 with valine (V)]. Basal to these clusters are isolates from the states of Minnesota, Washington, and California. The relatedness of other U.S. and New York isolates suggests that viruses spreading locally could have been introduced to New York through a domestic route (fig. S1).

The second cluster was a smaller group that contained four isolates from Manhattan/NYC and one isolate from Queens/NYC. ZIP code information was available for three of the Manhattan cases, which were mapped to three different neighborhoods, further supporting community spread. Although most NYC cases are intermixed within this largely European clade, these results suggest that domestic introductions may have also been a source of early community spread within NYC.

SARS-CoV-2 is the cause of one of the largest noninfluenza pandemics of this century. Sequencing of SARS-CoV-2 isolates from 84 patients diagnosed with COVID-19 at one of the largest healthcare systems in NYC during the first weeks of March 2020 provides insights into the origin and diversity of this new viral pathogen in the region. We found clear evidence for multiple independent introductions into the NYC metropolitan area from different regions globally, as well as from other parts of the United States. Our data indicates that early introductions by cases that were identified on the basis of their known travel histories did not seed the larger community clusters, suggesting that their early quarantine and hospitalization were effective in curtailing further spread. With increased testing, we observed the emergence of community-acquired infections. Most of these were caused by viral isolates derived from clades that were circulating in Europe, likely reflecting local transmissions from undetected introductions.

A limitation of our analysis is the relatively small number of global isolates from cases identified in the first weeks of March 2020, which means that our model relies on inferences based on available background sequences at that time. Thus, some of our inferences may change as more complete and representative SARS-CoV-2 sequences become available. Moreover, because global sequencing efforts are disparate, the fraction of sequences available by region or country is not necessarily representative of the number of cases reported for each of these regions. As shown by our estimates from different methods and molecular clock models, the time of the most recent common ancestor (tMRCA) estimates and precision can vary even with the same data. Last, in the absence of detailed epidemiological data on travel history and contacts, we were not able to associate periods of untracked transmissions with any specific regions or countries.

Taken together, we provide a first analysis of the SARS-CoV-2 viral genotypes collected from patients seeking medical care in the NYC metropolitan area. We found that NYC, as an international hub, provides not only a snapshot of the diversity of disease-causing SARS-CoV-2 at the global level but also informs on the dynamics of the pandemic at the local level. Future studies as well as additional sequencing are needed to define viral phenotypes and further explore the evolution of the SARS-CoV-2 epidemic in NYC, as well as to assess the impact of the public health measures on community transmission.

## Supplementary Materials

science.sciencemag.org/content/369/6501/297/suppl/DC1 Materials and Methods Figs. S1 to S3 Tables S1 and S2 References (*11*–*25*) MDAR Reproducibility Checklist Data Files S1 and S2

## Appendix

View/request a protocol for this paper from *Bio-protocol*.
